# Supplementary material for: Comparison of Osteoconductive Ability of Two Types of Cholesterol-Bearing Pullulan (CHP) Nanogel-Hydrogels Impregnated with BMP-2 and RANKL-Binding Peptide: Bone Histomorphometric Study in a Murine Calvarial Defect Model
Source: Int J Mol Sci. 2023 Jun 5;24(11):9751. doi: 10.3390/ijms24119751 (PMC10253458; doi:10.3390/ijms24119751)
Supplement: Supplementary file 1 [file ijms-24-09751-s001.zip › ijms-2381892-supplementary.pdf]

Supplementary Materials

Materials and Methods:

The degradation behavior of the CHP-OA nanogels was analyzed. The nanogels were immersed in three buffer solutions of pH 6.0, 7.4, and 8.0, and samples were collected over time up to day 66. The amount of degraded nanogels was determined from the supernatant after centrifugation at 4 °C, 3000 G, 10 mins. Quantification of degraded nanogels was analyzed by the phenol-sulfuric acid method [1], using a UV-visible spectrophotometer (visible light at 490 nm; Cary 60 UV-vis, Agilent, CA, USA). The degradation rate was calculated as the percentage of the amount of nanogels released after immersion in buffer solutions divided by the amount of nanogels initially immersed as follows:

Nanogels degradation rate (%) = (Amount of nanogels released into solution)/(Amount of initial solid nanogels) X 100

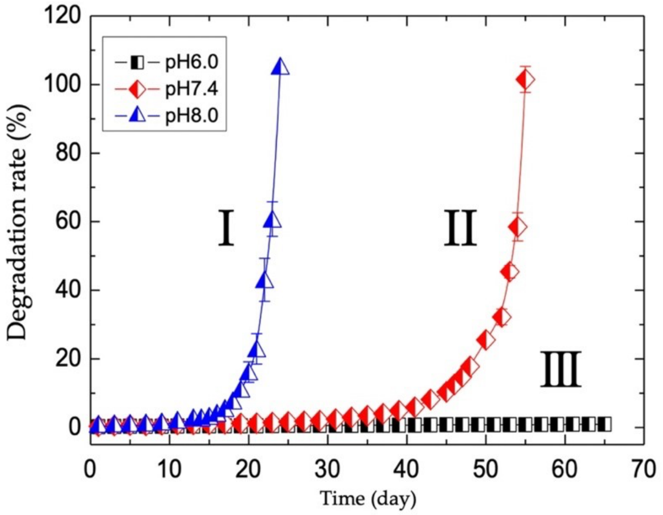

Supplementary Figure S1. Degradation behavior of CHP-OA nanogels in different pH buffers: CHP-OA nanogels in buffers of pH 8.0, 7.4, and 6.0, respectively. I: pH 8.0, II: pH 7.4, Ⅲ: pH 6.0, Time: the number of days immersed in buffer solutions.

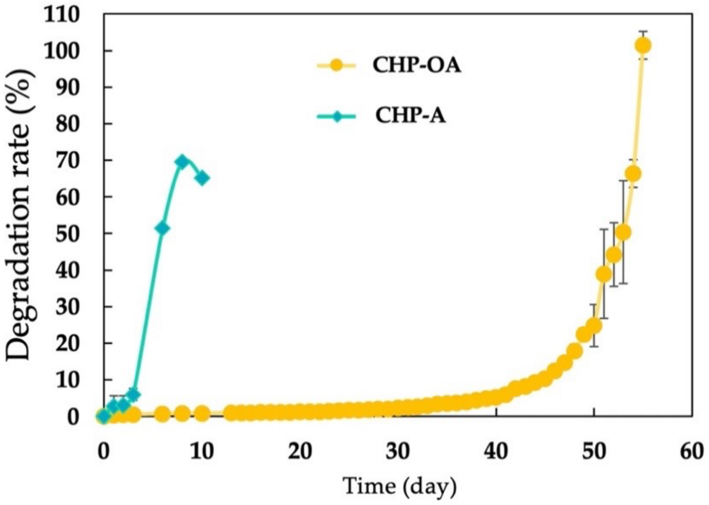

Supplementary Figure S2. Time-course study of the degradation rate of CHP-OA nanogels and CHP-A nanogels in pH 7.4 buffer solution. We performed these experiments on both nanogels simultaneously. The degradation data of CHP-A nanogels were reprinted/adapted from Xie et al. [2] to compare with those of CHP-OA nanogels. The detailed methods for measuring the degradation rate are described in the Materials and Methods section.

References

1. Dubois, M.; Gilles, K.; Hamilton, J.; et al. Colorimetric Method for Determination of Sugars and Related Substances. *Anal Chem*. **1956**, 28 (3), 350-356. https://doi.org/10.1021/ac60111a017.
2. Xie, C.; Satake-Ozawa, M.; Rashed, F.; Khan, M.; Ikeda, M.; Hayashi, S.; Sawada, S.; Sasaki, Y.; Ikeda, T.; Mori, Y.; Akiyoshi, K.; Aoki, K. Perforated Hydrogels Consisting of Cholesterol-Bearing Pullulan (CHP) Nanogels: A Newly Designed Scaffold for Bone Regeneration Induced by RANKL-Binding Peptides and BMP-2. *Int J Mol Sci.* **2022**, *23*, 7768. https://doi.org/10.3390/ijms23147768.
